# Barriers and facilitators for recruiting and retaining male participants into longitudinal health research: a systematic review

**DOI:** 10.1186/s12874-024-02163-z

**Published:** 2024-02-22

**Authors:** Danielle J. Borg, Melina Haritopoulou-Sinanidou, Pam Gabrovska, Hsu-Wen Tseng, David Honeyman, Daniel Schweitzer, Kym M. Rae

**Affiliations:** 1 Pregnancy and Development Group, Mater Research – The University of Queensland, Aubigny Place, South Brisbane, 4101 Australia; 2 Faculty of Medicine, University of Queensland, Herston, 4006 Australia; 3 Experimental Melanoma Therapy Group, Faculty of Medicine, The University of Queensland, Herston, 4006 Australia; 4 Indigenous Health Group, Mater Research Institute – The University of Queensland, Aubigny Place, South Brisbane, 4101 Australia; 5 Stem Cell Biology Group, Mater Research Institute – The University of Queensland, Translational Research Institute, 37 Kent Street, Woolloongabba, QLD 4102 Australia; 6 Library, University of Queensland, St Lucia, 4072 Australia; 7 Department of Neurology, Mater Health, South Brisbane, 4101 Australia

**Keywords:** Health research, Longitudinal study, Recruitment facilitators, Men, Gender, Study retention

## Abstract

**Background:**

Successfully recruiting male participants to complete a healthcare related study is important for healthcare study completion and to advance our clinical knowledgebase. To date, most research studies have examined the barriers and facilitators of female participants in longitudinal healthcare-related studies with limited information available about the needs of males in longitudinal research. This systematic review examines the unique barriers and facilitators to male recruitment across longitudinal healthcare-related research studies.

**Methods:**

Following PRIMSA guidelines, MEDLINE, Embase, CINAHL and Web of Science databases were systematically searched using the terms recruitment and/or retention, facilitators and/or barriers and longitudinal studies from 1900 to 2023 which contained separate data on males aged 17–59 years. Health studies or interventions were defined longitudinal if they were greater than or equal to 12 weeks in duration with 3 separate data collection visits.

**Results:**

Twenty-four articles published from 1976–2023 met the criteria. One-third of the studies had a predominantly male sample and four studies recruited only male participants. Males appear disinterested towards participation in health research, however this lack of enthusiasm can be overcome by clear, non-directive communication, and studies that support the participants interests. Facilitating factors are diverse and may require substantial time from research teams.

**Conclusions:**

Future research should focus on the specific impact of these factors across the spectrum of longitudinal health-related studies. Based on the findings of this systematic review, researchers from longitudinal health-related clinical trials are encouraged to consider male-specific recruitment strategies to ensure successful recruitment and retention in their studies.

**Registration:**

This systemic review is registered with the PROSPERO database (CRD42021254696).

**Supplementary Information:**

The online version contains supplementary material available at 10.1186/s12874-024-02163-z.

## Background

Recruitment into and continued participation of participants in clinical research provides continued challenges for researchers [1]. This is particularly true for participants who identify as men, likely due to the social roles and norms gender plays in society [2]. Recruitment is time-consuming, expensive, and the involvement and retention of male participants, as part of longitudinal healthcare studies, can be enormously demanding. It is likely that social constructs related to men such as cultural perceptions and health-seeking behaviour [3], have contributed to the challenges of male participant recruitment in healthcare-related research. However, these specific barriers have not been systematically investigated as part of previous clinical-research studies. Previous studies have explored the attitudes, beliefs and knowledge of the public towards research and research participation, focusing on clinical trials [4]. The public’s willingness to participate may be informed by a favourable attitude towards researchers, comprehension of the trial rationale, or the specific clinical circumstances (e.g., having a non-fatal disease with no known cure, being healthy, or critically ill with a limited chance of survival) [4]. It is important to note that, research findings often require a considerable amount of time to transition into clinical practice, and it is essential to educate the public about this process to encourage their participation in studies, ultimately advancing the progress of research. Given the time lag between findings resulting from healthcare research studies to healthcare implementation, an important component of participation is to enhance the public understanding of healthcare related research studies [1, 4, 5].

There are a range of population groups among whom it can be particularly challenging to recruit as part of longitudinal health studies and can include disadvantaged, minority and vulnerable members of the community. While others have systematically reviewed the recruitment and retention of participants in health studies related to conditions including cancer, dementia, and HIV, as well as studies involving vulnerable populations [6–8], less is known about recruitment and retention of male participants as part of longitudinal health-related studies. This highlights the need to address recruitment issues in a broad spectrum of healthcare-related research studies for males.

Although several healthcare-related studies have examined recruitment of male participants across diverse populations groups, there is limited research identifying barriers and facilitators associated with overall male recruitment into healthcare-related studies. Notably, there are male-specific clinical changes across healthcare that can influence interest in related research [9]. Life expectancy is lower in males [3], especially those aged over 50 years, who often experience a greater disease burden [10]. Although previous studies demonstrate that men are disengaged with healthcare services, it is now recognised that males engage willingly and effectively with healthcare that recognise the preferences of males [10–12]. Previous literature have investigated methods of improving male recruitment to health behaviour research [13]. Indeed, sex was an important determinant of health-risk and health-promoting behaviours [14], with males being more likely to perform high-risk behaviours including smoking, unhealthy eating, excess alcohol consumption, and physical inactivity [3, 15] and despite this, remained less likely to seek medical and psychological help when needed [16] or to participate in health-promotion programs [17]. Maher et al., detailed that males only comprise about 20% of health behaviour research participants, in mixed sex studies [18], contributing to a lack of evidence on how to increase the uptake of health-promoting behaviours for males [19]. These findings highlight the need for highly effective, male-specific methods to assist recruitment and retention in research studies in line with current best practice and guidelines.

Effective long-term recruitment methods to enable and facilitate male recruitment in longitudinal healthcare research consistently demonstrate strategies should be tailored for age, interests, and sex. To facilitate the effective recruitment of men into research, different recruitment methods for different age groups of either sex can be effective [11, 20]. Younger males may be recruited through online social network platforms including Facebook [21, 22], which is less effective in elderly males [23]. While elderly men would be more likely to participate if referred to the study by their affiliated health service provider, media coverage or mass mailings [11]. Facebook, in particular, is more effective at recruiting participants than any of the other social media platforms combined [21]. As of October 2020, more males globally (57%), use Facebook than females (43%) [24]. Yet, a recent systematic review of recruitment using Facebook, found little evidence of its effectiveness in recruiting participants of either sex aged over 35 years [22], highlighting that social media strategies were ineffective. Tolmie et al., reported that the need for ongoing health monitoring for older participants was the most important recruitment and retention motivator, in addition to fostering positive relationships between staff and participants, and communicating the studies progress to recruits [25].

The difficulty of recruiting and retaining males in research studies can adversely affect the statistical study power and generalisability of study findings, and in particular, those studies involving a longitudinal design which consequently affects the applicability of results to the male population [26, 27]. While sex (male) and gender (men) constructs are important considerations in society and within health, to date, the terms male and men are often used interchangeably in health literature. For these reasons, this systematic review has reviewed published studies that consider male participation, recognising that the terms male and men, most often refer to the biological construct of male sex. This review has focused on health research or health interventions that are using a longitudinal study design. The main outcome was to identify specific barriers and facilitators of male recruitment and retention as part of longitudinal research-related studies. Findings have the potential to inform future development of patient-centred and evidence-based strategies to enhance recruitment into longitudinal health-related studies for men.

## Methods

This systematic review protocol was registered with PROSPERO database (University of York Centre for Reviews and Dissemination) (CRD42021254696) and complies with reporting guidelines from the Preferred Reporting Items for Systematic Reviews and Meta-analysis (PRISMA) statement [28].

### Study identification

Studies published in English without date restrictions were identified through systematic searching. There was no limit applied to the dates of publications, in order to explore the full breadth of the literature surrounding the topic and determine strategies used that remain relevant in this current time. The databases MEDLINE (Ovid, 1946 to present), Embase (Embase.com, 1947 to present), CINAHL (EBSCO, 1981 to present), and Web of Science (Clarivate Analytics, 1900 to present) were searched on 20 October 2020. The searches were updated on 21 October 2021 and 11 November 2023 to determine any additional publications during the 2020–2023 period. The MEDLINE search strategy was translated for the other databases using the Polyglot Search Translator [29]. Specific search terms used (see Supplementary File 1) included recruitment and/or retention, facilitators and/or barriers, and longitudinal studies. Here, longitudinal research was defined as a study with a minimum of three repeated study visits, or research data collections over a time greater than or equal to 12 weeks. Search terms were used with Boolean operators “AND” and “OR” as connective devices within the search strings. Where appropriate and possible, search terms were truncated (*) to retrieve multiple variations of a word.

All retrieved articles, excluding duplicates, were exported into Covidence [30] to facilitate the screening process. Identified studies were screened by two independent reviewers from the review team (DJB, MH-S, PG, HWT, DH, DS) to identify eligible studies. Studies were assessed for inclusion based on screening of title and abstracts. A third independent reviewer (KMR) reviewed conflicts. Full text papers were retrieved and assessed by two team members according to the inclusion and exclusion criteria with conflicts reviewed by third independent reviewer (KMR). The reference lists and citing articles of included studies and relevant reviews and systematic reviews were further hand-searched for further potential papers for inclusion.

### Inclusion criteria

#### Types of participants

This review includes any participant who identifies as male (biological sex). Where the included publication does not make it clear if this is the biological definition of males, or the gendered definition of men, the assumption has been made that this refers to those who are biologically male, and therefore included. Male participants between the ages of 17 – 59 years were included. We chose 17 years of age as the lower age limit to encompass studies involving adult males that did not require parental consent. The upper limit of 59 years was set to eliminate recruitment of older populations, as previous research has covered participation of older populations extensively and we aim to investigate factors influencing the involvement of younger individuals. Studies covering a broader age range were considered only if they provided age-specific data. Male parents, who were consenting on behalf of their child into a longitudinal health study or intervention were included. Parents who consent on behalf of a child are often needed to take part in certain aspects of the study; however only studies that specifically identified parental sex were included. Studies that included females or indeterminate sex were reviewed however these studies were only included if recruitment and/or retention of males and men were reported separately. Likewise, studies of parents and child or family studies were included if they reported recruitment and/or retention of male parents separately.

#### Types of studies

Any cross-sectional, longitudinal, survey, experimental, program evaluation studies or studies involving qualitative or mixed methods that intentionally (i.e., stated a priori) or incidentally (i.e., noted as a posteriori) included detailed commentary or analysis on the recruitment and/or retention of male participants in a longitudinal health intervention or health research study, with the requirement that this commentary offered informative data rather than a generalised statement about participant recruitment or retention.

#### Types of exposures/interventions

This review excluded studies focused on Alzheimer’s/dementia [31], cancer [32], HIV [33] and illegal drugs [34] due to the wealth of existing systematic review literature. Studies focusing on fathers with young children in early childhood health intervention research were excluded due to a recent systematic review [35].

Any other health research program or health intervention was included, provided enrolled male participants had data collected on a minimum of three separate occasions over a period of greater than or equal to 12 weeks. Longitudinal studies that were less than 12 weeks in duration or had less than 3 study visits or data collections were excluded. A health intervention was defined as any study aimed at improving specific health behaviours or outcomes.

#### Types of outcome measures

Studies were included if they identified specific strategies for recruiting and retaining male participants into longitudinal research and longitudinal clinical practice and if findings were analysed, reported, or discussed separately.

### Exclusion criteria

#### Study population

Studies on recruitment, retention, barriers and facilitators of vulnerable populations, males < 16 years and males > 60 years of age were excluded. Vulnerable male populations were defined as socioeconomically disadvantaged populations or racial and ethnic minorities (including Indigenous and First Nations people). Due to the cultural, economic and other differences that a review of these communities would likely identify, it was deemed to be appropriate for these populations to be reviewed separately in the future.

#### Study topic

Conference abstracts, review or systematic review papers, incomplete studies including study protocols, and grey literature were excluded from this review. Articles were excluded at any time in the screening process, if they did not (1) examine views or include discussions that considered retention, barriers, or facilitators for research/interventions; (2) include male specific data, and only discussed female participants; (3) determine the participant sex or (4) focus on participant recruitment or retention as part of the health research/intervention.

### Data extraction

Data extraction was performed by three members of the review team (DJB, PG, MH-S) and reported narratively. Extraction was cross-checked for accuracy and consistency by one other reviewer (either DS or KMR). The following information was extracted from each included study: publication information, study aim, methods (i.e., participants, procedures, demographics), recruitment of male participants, retention of male participants, timing of data collection, and types of data collected from male participants. Reported barriers and facilitators to support male recruitment and retention was extracted.

### Quality assessment

A quality assessment check is usually undertaken in a systematic review that pertains to a review that assesses the individual results of a group of specific studies. As this review assesses the barriers and facilitators to recruitment and/or retention methods, there was no need for a quality check of the included studies.

## Results

**Fig. 1:**
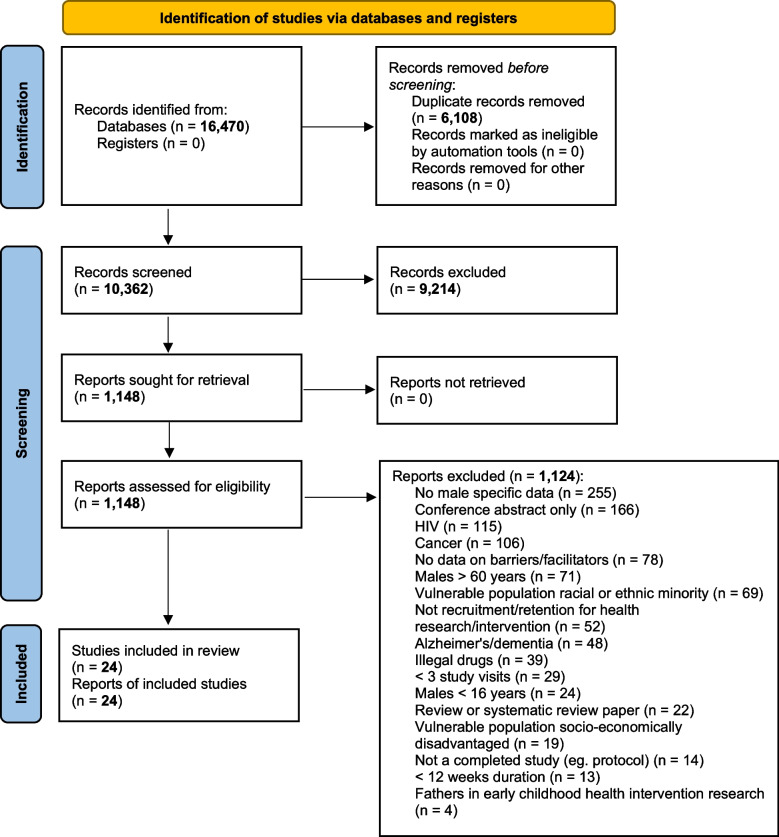
PRISMA diagram depicts the search, screening, eligibility and inclusion results

The database searching and the forward and backward citation checking yielded 16,457 and 13 papers respectively (16,470 total). 6,108 duplicates were removed resulting in 10,362 articles available for screening (Fig. 1). Of these, 9,214 studies did not meet the inclusion criteria based on titles and abstract screening and resulted in 1,148 full-text studies selected for further screening (Fig. 1). A total of 1,124 studies were then excluded with 255 having no male specific data, 166 conference abstracts, 115 HIV related research, 106 cancer related research, 78 studies had no included data on barriers or facilitators, 71 studies with a focus on males > 60 years, 69 studies from racial or ethnic minority, 52 studies were unrelated to health recruitment and retention, 48 Alzheimer’s or dementia research, 39 related to illegal drugs, 29 papers were studies with less than 3 study visits, 24 papers were males < 16 years of age, 22 systematic review/review papers, 19 focused on socioeconomically disadvantaged populations, 14 uncompleted studies/study protocol, 13studies were < 12 weeks duration, and 4 fathers in early childhood interventions (Fig. 1).

A total of 24 articles remained and the data was extracted and included in this review. The oldest of these studies was published from 1976 [36] and the most recent, 2023 [37, 38]. All of the included studies were conducted in Western countries except Cheraghi et al., which was based in the Middle East [39] and Schilling et al., which was based in India [37]; two were located in United Kingdom [40, 41], two in France [42, 43], one in Finland [44], one in Sweden [45], one in The Netherlands [46], one study across combined European nations [29], one in Germany [47] ten in North America [36, 48–56] and three in Australia [38, 57, 58] and are described in Table 1. Participant characteristics varied with study focus including participants with specific health conditions, such as overweight [41, 57], having an occupational injury [40, 41], having visited a sexually transmitted infection clinic [46], or being treated for a psychological disorder [44, 50, 53], COVID related issues [37, 54], or habits such as alcohol abuse and smoking [56]. Some studies recruited participants from specific subgroups, including veterans [36], workers of an electricity company [42] and people that had attended a spouse abuse abatement program [50]. All twenty-four studies met the inclusion criteria for age. One of the studies was a family cohort study that recruited families of children with cystic fibrosis and congenital heart disease and required participation of both parents [51].

**Table 1:** General study characteristics. Studies are listed alphabetically

*Author / Year*	*Study Name (if identified)*	*City (or State/ Province/ Region), Country*	*Study Aim/s*	*Study Design*	*Clinical field*	*Type of data collected*
Amin et al. 2023 [38]	Substudy of PREVENT-ADPKD	Sydney, Wollongong, New Lambton Heights, Gosford, Perth, Australia	To determine the experiences of participants in a long-term trial Identify factors influencing reasons to enrol, remain, and adhere to trial procedures Develop preliminary recommendations for improving future clinical trials	Randomized control trial	Kidney Disease	Factors hypothesized to influence participant recruitment and retention (ranked the utility and difficulty of individual components), overall global satisfaction score, Qualitative: Semi structured Interview (explored expectations and motivations before enrolling, adequacy of informed consent, overall experience, perceived adherence, interactions with study staff and recommendations for future trial design
Cheraghi et al. 2021 [39]	Tehran Lipid and Glucose Study (TLGS) [59]	Tehran, Iran	Clarify factors associated with non-participation in the TGLS cohort Identify sub-groups who are likely to refuse participation and improve retention strategies for these groups	Longitudinal cohort study	Diabetes and non-communicable diseases (NCDs)	Protocol [59] and this paper include data on prevalence of NCDs, risk factors for NCDs, intervention data for intervention undertaken by specific target groups of cohort including schoolchildren, women, high-risk groups. Interventions focused on lifestyle modifications; diet, smoking and physical activity
Crichton et al. 2012 [57]	-	Adelaide, Australia	Evaluate the recruitment process, retention of participants and challenges faced in a dairy intervention trial Provide strategies to combat the difficulties of running long-term dietary intervention trials	Randomised controlled dietary intervention	Obesity	Height, weight, blood pressure, health and dietary questionnaires, anthropometry, and blood samples taken at each visit (cardiometabolic and biochemical assessment), arterial compliance, cognitive assessment and mental health assessment
Goldberg et al. 2006 [42]	GAZ and ELectricité (GAZEL) Cohort Study	Saint- Maurice, France	Determine the socioeconomic, lifestyle, and health factors associated with response to annual mail questionnaires	Longitudinal cohort study	-	Questionnaires on health status, lifestyle, socio-economic and occupational factors
Gourash et al. 2022 [48]	The Longitudinal Assessment of Bariatric Surgery (LABS)	New York, Greenville, Fargo, Portland, Pittsburgh, Seattle, The United States of America	Determine short-term and long-term safety of bariatric surgery Evaluate psychosocial, quality of life, health economics of obesity, co-morbidities and bariatric surgery	Prospective cohort study	Obesity	Blood draw, urine sample, physical measures: anthropometrics, blood pressure, corridor walk, activity monitor and diary, sociodemographic, psychologic, behavioural data
Griffith Fillipo et al. 2022 [49]	-	Throughout the United States of America	Determine the optimal incentive strategy for study retention	Remote and sequential multiple assignment randomized trial	Psychology	Demographics, Major Depressive episode Screener, Patient Health Questionnaire-9 (PHQ9), The Social Life and Family Life Scales of the Sheehan Disability Scale, 7-item Generalized Anxiety Scale (GAD-7) the NIAAA Alcohol Screening Test, IMPACT assessment of main and psychosis, exit survey
Green et al. 2018 [40]	Cambridge Centre for Ageing and Neuroscience (Cam-CAN) study [60]	Cambridge, United Kingdom	Determine the factors that affect response in epidemiological studies	Contemporary cross-sectional population-based cohort study	Epidemiology and Aging	The included study and its accompanying protocol paper [60] collect interview data related to recruitment and inclusion criteria, core cognitive neuroscience (MRI, MEG, cognitive testing), blood pressure, salivary sample and 3 fMRI
Hamberger et al. 2000 [50]	-	Wisconsin, United States of America	Identify reasons for participant dropout from a spouse abuse abatement program	Cohort study	Psychology	Race, education, age, marital status, employment status, alcohol abuse, referral status, police violence contact, drug activity, acting out, history of child abuse, witnessing violence, violence duration, violence level report
Irvine et al. 2017 [41]	-	Dundee, United Kingdom	Assess feasibility of trial for reduction of alcohol consumption in obese men	Community-based Intervention	Obesity	Alcohol consumption measures and body mass index
Janus et al. 1997 [51]	-	Toronto, Canada	Determine factors influencing family participation in a longitudinal study	Longitudinal birth cohort study	Cystic fibrosis, Congenital heart disease	Severity of chronic illness, diary completed by parents, developmental level, child temperament, family environment, Mother–Child relationship
Kannisto et al. 2017 [44]	Mobile.Net	Turku, Finland	Determine the dropout predictors from a mHealth-based trial and evaluate the effects of tailored short message service (SMS) text message constructed to encourage patient adherence	mHealth-Based Randomized Controlled Trial	Psychology	Participant’s quality of life (Q-LES-Q) and satisfaction with the treatment (CSQ-8)
Kelfve et al. 2017 [45]	Swedish level-of-living survey (LNU)/ The Swedish panel study of living conditions of the oldest old (SWEOLD)	Stockholm, Sweden	Determine how selective survey participation affects the sample composition, in addition to selective mortality of participants	Longitudinal cohort survey study	Aging	Both LNU and SWEOLD primarily use face-to-face interviews to gather data. The questionnaires used cover a broad range of topics, such as living conditions, family situation, health, health behaviours, and financial resources
Lee et al. 2009 [61]	The European Male Ageing Study (EMAS)	European cities: Florence (Italy), Leuven (Belgium), Lodz (Poland), Malmo ¨ (Sweden), Manchester (United Kingdom), Santiago de Compostela (Spain), Szeged (Hungary) and Tartu (Estonia)	Examine aspects of aging in men	Prospective cohort study	Aging	Questionnaire: assess quality of life, depressive symptoms, Adverse Life Events Scale, Physical Activity Scale, International Prostate Symptom Score, previous surgical procedures, sexual function questionnaire. Screening: Physical and cognitive performance, Anthropometry, Calcaneal ultrasound, Food diary, blood samples
Limmroth et al. 2023 [47]	PROmyBETAappGame	Multiple Locations, Germany^a^	Investigate medication-taking behaviour in patients with multiple sclerosis receiving interferon β-1b for 1 year and to provide additional information on patient reported outcomes. To test the feasibility of gamification (using cognitive training tool PEAK) to incentivise patients to remain committee to the study	Prospective and retrospective, noninterventional observational cohort study	Multiple sclerosis	Demographic data: age gender, injection-related data: date/time of injection, injection speed and injection depth (collected from the app or manually recorded), self-reported measures: health-related quality of life, EuroQol-5-Dimension 5-Level questionnaire, Treatment Satisfaction Questionnaire of Medication, questionnaire on satisfaction with the BETAPLUS, BETACONNECT and the app via a service questionnaire
Markanday et al. 2013 [58]	Geelong Osteoporosis Study	Geelong, Australia	Investigate sex-differences in non-participation at baseline of the Geelong Osteoporosis Study (GOS)	Prospective cohort study	Osteoporosis	Reasons for not participating
Méjean et al. 2014 [43]	NutriNet-Santé Study	Paris, France	Evaluate relationships between participation motives and sociodemographic, health, and lifestyle characteristics of participants in cohort designed to identify nutritional risk or protective factors for chronic diseases	Web-based, Prospective cohort study	Nutrition	Questionnaires assessing dietary intake, physical activity, anthropometrics, lifestyle, and socioeconomic conditions along with health status
Oleske et al. 2007 [52]	-	3 Midwestern states^b^, United States of America	Determine influence of demographics, health, and job factors in continued participation of employed persons in a longitudinal intervention study for work-related low-back disorders	Randomized clinical trial	Work-related low-back disorders	Self-reported measures: Back pain frequency and how bothered participants were by pain, back pain disability, physical health, mental health, neurogenic symptoms, psychological job strain / Measures: height, weight, body fat percentage, waist and hip circumference, body mass index
Olmos-Ochoa et al. 2019 [53]	-	Los Angeles, United States of America	Identify barriers to study participation and retention, in two modes of intervention for serious mental illness	Cohort study	Psychology	Barriers to physical activity and healthy eating
Pogue et al. 2022 [54]	-	North and Central Texas, United States of America	To investigate the impact of the COVID-19 pandemic on mental health	Longitudinal study	Psychology	Depression (PHQ-8), posttraumatic stress symptoms (Posttraumatic Diagnostic Scale-5), anxiety (GAD-7), resilience (10-item Connor-Davidson Resilience Scale), coping strategies (brief COPE), physical activity (International Physical Activity Questionnaire), personality (10-item Personality Inventory), COVID stigma, vaccine hesitancy, COVID-19 impact (COVID Impact Scale), COVID fear (COVID Fear Scale), workplace demographics
Rose et al. 1976 [36]	Normative Aging Study of the Veterans Administration	Boston, United States of America	Determine the non-pathological aspects of aging	Longitudinal health cohort	Aging	Age, social class, geographic stability and health
Schilling et al. 2023 [37]	Data4Life Consortium	Pensieve Health (Mumbai), Hande Hospital (Panvel, Navi, Mumbai), ACUMDX Laboratory and Research Center (Ghatkopar, Mumbai, Kota, Jodhpur in Rajasthan) and Sun Diagnostics (Ghatkopar, Mumbai), India	Investigate (via digital recruitment and data collection methods) the long-term effects of COVID-19 in India	Observational, longitudinal study	Infectious disease	Demographics, participant feedback, medical and COVID-19 history, physical measurements, social connections, isolation risks, depression (PHQ-9), anxiety (GAD-7), activity levels (IPAQ-SF), asleep patterns (PSQI), alcohol, tobacco, dietary habits
Snow et al. 2007 [55]	The Lung Health Study (LHS)	Minneapolis, United States of America and Winnipeg, Canada	Examine the impact of smoking cessation coupled with the use of an inhaled bronchodilator on chronic obstructive pulmonary disease	Randomized, controlled clinical trial	Chronic obstructive pulmonary disease	Demographic variables, alcohol intake, body mass index (BMI), smoking-related variables, past and present illness, lung function, and social support variables
Ullmanet al. 1998 [56]	Newcomb [62]	Los Angeles, United States of America	Develop models that differentiate eager, reluctant, and nonresponding participants using participants’ demographics, personality, and drug use characteristics	Longitudinal cohort study	Psychosocial health and Substance use	The included paper and its accompanying protocol [62] collect data on use of monetised incentives for longitudinal recruitment, participant demographics, drug and alcohol intake, personality and attitudinal traits, including support of science/medicine and social conformity
vanWees et al. 2019 [46]	Mathematical models incorporating Psychological determinants: control of Chlamydia Transmission (iMPaCT)	Amsterdam, Hollands Noorden, Kennemerland, and Twente, the Netherlands	Identify predictors of non-response and loss to follow-up in longitudinal sexual health study	Longitudinal cohort study	Chlamydia Transmission, Sexually transmitted infections (STI)	Data on sexual behaviour, psychological determinants and chlamydia infections. Participants were tested for chlamydia using nucleic acid amplification tests at enrolment at the STI clinic and through a self-sampling kit sent to a laboratory at six-month follow-up

^a^ClinicalTrials.gov NCT03808142 lists multiple locations in Germany, however locations with principal investigators are not listed

^b^Midwestern states are not detailed

Of the included studies, 20 had male and female participants [37–40, 42–49, 51–58], with a number of these studies having a predominantly male sample [42, 52, 53, 58]. Four studies recruited only male participants [36, 41, 50, 61] (Table 2). The included studies with mixed sex either described male and female characteristics separately or clearly stated that there were no significant differences in recruitment and retention based on sex. All included studies used a minimum of three study visits or data collection, and the maximum number of study visits or data collections was 95 visits [41] and one study had up to 300 interactions with participants [44]. The minimum study length of included studies was 16 weeks [50] and the maximum study duration was 43 years [45]. All included studies collected demographic data [36–58, 61].

**Table 2:** Study duration, number of study visits, percentage of male participants of the study, and details of recruitment and retention numbers (n.d indicates that no data was available in the published literature)

*Author / Year*	*Study duration*	*Study status at time of publication (Rec Recruitment, Ret Retention, F Follow-up)*	*Number of Study visits/ contacts*	*% of male participants in the study*	*Total number of male participants recruited*	*Total number of male participants retained*	*% male participants retained*
Amin et al. 2023 [38]	3 years	F	9^a^	50%	*n* = 93; parent study	*n* = 75; Questionnaire *n* = 19; Interview	80.1% Questionnaire 16.1% Interview
Cheraghi et al. 2021 [39]	20 years	Ret & F	5	42.39%	*n* = 4,395	n.d^#^	55% > 60 years 67% 40–59 years 57% 20–39 years
Crichton et al. 2012 [57]	1 year	Rec, Ret & F	3	27%	*n* = 20; 18–71 years	*n* = 10	50%
Goldberg et al. 2006 [42]	11 years	F	11	73%	*n* = 8,550; 40–45 years *n* = 6,277; 45–50 years	n.d^*^	n.d
Gourash et al. 2022 [48]	5 years	Rec, Ret & F	6	21.4%	*n* = 527	n.d^*^	n.d^*^
Green et al. 2018 [40]	Study launched in 2010^b^	Rec	3^c^	43.7%	*n* = 3,315	*n* = 1,172	35.4%^*#*^
Griffith Fillipo et al. 2022 [49]	13 weeks	Ret & F	3	14%	*n* = 19; low incentive *n* = 11; high incentive	*n* = 30	100%
Hamberger et al. 2000 [50]	Recruitment duration: 6 years 2 months, Study duration: 16 weeks	Rec, Ret & F	16	100%	*n* = 534	*n* = 150; 25–34 years *n* = 49; < 25 years *n* = 76; ≥ 35 years	28% 25–34 years 9.2% < 25 years 14.2% 35 years
Irvine et al. 2017 [41]	5 months	Rec, Ret & F	96	100%	*n* = 69	*n* = 59	85.5%
Janus et al. 1997 [51]	4 years	Ret	4	50%	*n* = 209	*n* = 135	64.6%
Kannisto et al. 2017 [44]	1 year	Rec, Ret & F	24-300^d^	49.17%	*n* = 560	*n* = 227	41.0%^e^
Kelfve et al. 2017 [45]	43 years	F	7	15.2%	*n* = 172	*n* = 134^f^	41.4% 77–87 years
Lee et al. 2009 [61]	6 years	Rec	3	100%	*n* = 3,963	*n* = 3,369	38.7% 40–49 years 45.0% 50–59 years 43.2% 60–69 years 34.4% > 70 years
Limmroth et al. 2023 [47]	1 year	Rec, Ret & F	5	34%	*n* = 21	*n* = 21	100%
Markanday et al. 2013 [58]	10 years	Rec	3^g^	50.8%	*n* = 2,296	*n* = 1,540	67.1%
Méjean et al. 2014 [43]	10 years	Rec & Ret	11-120^h^	24.1%	*n* = 3,929	*n* = 1,531	24.1%
Oleske et al. 2007 [52]	1 year	F	5	79.3%	*n* = 360	*n* = 168	83.6% 47.1 ± 6.7 years
Olmos-Ochoa et al. 2019 [53]	6 months	Rec, Ret & F	24 or 30^i^	Overall Study: 93.9% Sub Study: 83.3%	*n* = 260 *n* = 40	n.d^j^	n.d^*^
Pogue et al. 2022 [54]	2 years	Rec, Ret, F	6	25.5%	*n* = 324	n.d^k^	n.d^k^
Rose et al. 1976 [36]	12 years	Rec & Ret	n.d^l^	100%	*n* = 2,280	*n* = 2,028	88.9%
Schilling et al. 2023 [37]	1 year	Rec & Ret	12	74.02%	*n* = 4,371	n.d^*^	n.d
Snow et al. 2007 [55]	11 years	Ret & F	6	62.4%	*n* = 3,327	*n* = 2,749	82.6%
Ullman et al. 1998 [56]	16 years	Rec & Ret	4	37.6%	*n* = 207	*n* = 201	97.1%
vanWees et al. 2019 [46]	2 years	F	4	19%	*n* = 324	*n* = 206	63.6%

^*#*^Odds ratio reported only for male participants retained [39] or division of age groups [59]

^*^Data not divided between those identifying as men and those who identify as women

^a^Study protocol of PREVENT ADPKD [63], substudy visits not specified [38] but is assumed 2 (questionnaire, interview) in a 16 month period

^b^[40] and study protocol [60]. Study launched reported on study website [64]

^c^Protocol paper describes 3 Stages after recruiting a population-based cohort [60], not described in [40]

^d^Study contact were text messages the amount, timing and frequency of SMS text messages were decided by participant [44]

^e^Completers of final postal survey

^f^Last wave (wave 5) of the study (77 – 87 years old) completers of follow-up, no male specific data given for wave 1–4 [45]

^g^Protocol paper describes the use of census data at 1996, 2001 and 2006 [65]

^h^Yearly visits with an option to fill in a complementary questionnaire each month [43]

^i^Two studies were included WebMOVE and MOVE-SMI [53]

^j^Sub-study was randomly selected from WebMOVE and MOVE-SMI groups and not stratified by sex [53]

^k^Details only recruitment strategies [54]

^l^Due to its longitudinal nature, it is assumed this study has more than 3 visits in the 12-year reported period. No information on number of study visits or contacts was found in Rose et al. [36] or the associated protocol [66]

### Recruitment

Overall, all studies provided information on recruitment rates and 19 provided information on retention rates [36, 38–41, 43–47, 49–52, 55–58, 61] (Table 2). A variety of methods for male participant recruitment included advertising [36, 43, 54, 57], letters of invitation [39–43, 47, 52, 56–58, 61], selection of participants from larger cohorts [42, 43, 50, 53], or recruitment from hospitals or registers [37, 44, 48, 51, 54, 57] (Table 3). The most common method was sending letters of invitation, used in 11 out of the 24 studies, and yielded recruitment rates between 4.4% and 79.3% [47, 52]. Irvine et al., recruited participants through letter of invitation and time space sampling, and reported that time space sampling was difficult, time consuming and only yielded one participant per 11 field visits [23]. Snow et al., used multiple methods for recruitment, including recruitment from work sites and public sites, mass mailing, telephone, media, and referral methods and reported that mass mailing was the best method of these [55]. Rose et al., attributed their high recruitment rates to advertising and therefore people that agreed to participate had done so voluntarily and were more likely to be interested in the study and health interventions in general [36]. To maximise male participation, vanWees et al., adapted their recruitment methods to target male participants by raising awareness and a greater sense of responsibility in terms of male health through flyers or personalised invitations [46].

**Table 3:** Recruitment strategies with associated recruitment and retention rates of participants (n.d indicates that no data was available in the published literature)

Recruitment Method	Study	Recruitment Rate	Study Attrition Rate
**Advertising**	Crichton et al*.* 2012 [57]	35.7% of total participants (*n* = 30/84) via TV segment 31% of total participants (*n* = 26/84) via Newspaper	*n* = 20 males completed the study: 50% completers of 12-month follow up (*n* = 10/20) (10% Early drop out (*n* = 2/20), Dropouts during study (40% *n* = 8/20)
	Rose et al*.* 1976 [36]	n.d	11% attrition (*n* = 2,028/2,280). deceased *n* = 54 (2.4%); lost interest *n* = 103 (4.5%); moved away with no further participation *n* = 44 (1.9%); moved away with survey only participation *n* = 51 (2.2%)
	Méjean et al*.* 2014 [43]	Initial recruitment strategies for NutriNet-Santé Study included television, radio, national and regional newspapers, posters and Internet providing details about the study’s website. A total of 86,652 individuals were recruited (n.d for individuals identifying as male)	n.d
	Pogue et al*.* 2022 [54]	23.5% contacts made (*n* = 26,100/111,070) through social media. 58.6% views made from social media contact (*n* = 15,286/26,100). Recruitment rate of 73% for consent (1,987 attempts/1,452 consents were completed). 64.4% of participants were eligible (*n* = 1,279/1,452). Of these, 920 participants had demographic characteristics. Of those 3.5% (*n* = 32) were recruited via social media, of which 12.5% (*n* = 4) identified as male. Other media sources recruited a further 1.6% of participants (*n* = 15/920), of which 6.7% identified as male (*n* = 1/15). Word of mouth advertising recruited 11.2% (*n* = 103/920), of which 22.3% identified as male (*n* = 23/103)	63.4% attrition (*n* = 920/1,452) from consent to baseline sample where demographic characteristics were reported
**Invitation Following online intake assessment at clinic, via telephone or via non for-profit platform**	Van Wees et al*.* 2019 [46]	13,658 were eligible and invited. Overall, 12% of eligible individuals were recruited (*n* = 1,705), of these, 19% identified as male (*n* = 324)	From the total sample, 47.5% attended baseline visit (*n* = 810/1,705), 25.3% attended 3 week visit (*n* = 432/1,705), 24.3% attended 6 month follow-up (*n* = 416/1,705) and 20.2% attended 1 year follow-up (*n* = 344/1,705). Study attrition for both male and female participants by the 1 year follow up was 79.8%
	Griffith Fillipo et al*.* 2022 [49]	1,652 were assessed for eligibility via the Mental Health America online platform. No details are given about how eligible participants were invited. 215 participants were randomized. Of these 14% (*n* = 30) identified as male	52.8 attrition rate for the low monetary incentive group and 54.1% attrition rate in the high monetary incentive group by exit survey at week 12, study end. While males were more likely to end participation early compared to females, no further information on the numbers are given
**Letter of Invitation**	Crichton et al*.* 2012 [57]	84 responders from advertising. Interested potential participants were invited to an information session and pre-study screening. 71 participants screened and deemed eligible (84.5%; n.d for individuals identifying as male)	*Reported above*
	Cheraghi et al*.* 2021 [39]	All family members were invited for baseline measurements *n* = 15,005 individuals agreed to participate (≥ 3 years of age, n.d for individuals identifying as male)	39.6% of entire cohort after 5th follow up examination *n* = 10,368 individuals participating in current study. Of these *n* = 4,395 (42.4%) were male (*n* = 1,650 for intervention, *n* = 2,745 for control)
	Goldberg et al*.* 2006 [42]	GAZEL Study had 20,624 at baseline but 20,328 included in this study. 72.9% of participants identified as male (*n* = 14,827)	0.4% attrition rate over 12-year follow up due to leaving the company (*n* = 60), or leaving the study (*n* = 3) 87.2% of participants retained after first annual mail questionnaire returned in 1990 compared to baseline (n.d of numbers of participants or sex) 71.2% retained at the end of the study 12 years later in 2000 (n.d of numbers of participants or sex)
	Green et al*.* 2018 [40]	Invitation letter signed by GP and information sheet describing aims, nature and how to contact Cam-CAN study was sent to 7,616 eligible individuals. 35.2% (*n* = 2,680) consented. Of those, 43.7% were male (*n* = 1,172)	n.d due to single time point reported in this study
	Irvine et al*.* 2017 [41]	Letter received from GP inviting 47.1% of participants to take part in the study (*n* = 419 contacted out of 889 assessed for eligibility)	1.4% (*n* = 1 lost to follow up out of 69 consented participants)
	Lee et al*.* 2009 [61]	Average recruitment rate of 40%. Recruitment Rate via city and registry: Florence (Primary care; 59.9% *n* = 433/723), Leuven (Electoral; 37.9% *n* = 451/1189), Lodz (City registry; 48.4% *n* = 408/843), Malmö (Population; 44.6% *n* = 409/918), Manchester (Primary care; 37.2% *n* = 396/1064), Santiago (National register; 35.2% *n* = 406/1155), Szeged (Electoral; 24.1% *n* = 431/1789), Tartu (Primary care; 59.2% *n* = 435/735)	Average attrition rate of 8.5%. Attrition rate calculated at each site due to death or moved house: Florence (4.4% *n* = 414/433), Leuven (4.4% *n* = 431/451), Lodz (15.7% *n* = 344/408), Malmö (11.3% *n* = 363/409), Manchester (11.1% *n* = 352/396), Santiago (21.2% *n* = 320/406), Szeged (0% *n* = unknown/431), Tartu (0% *n* = unknown/435)
	Limmroth et al*.* 2023 [47]	Invitation sent as push messages to registered accounts for the app (*n* = 1,778). 79 patients consented (4.4%) of which 21 were male (1.18%). No gender specific data was given on the participants who withdrew or were excluded	0% attrition however, persistence (95%; *n* = 20/21 cf 90%; *n* = 19/21), compliance (primary end point) (97.7 *n* = 9/21 cf 95.4% *n* = 11.4/21) and adherence (90%; *n* = 19/21 cf 86% *n* = 18/21) were measured for each gender at 6 months compared to 12 months, respectively, from the prospective data set (above) and retrospective the data set: persistence (90%; *n* = 19/21 cf 86% *n* = 18/21), compliance (primary end point) (98.4% *n* = 6.7/21 cf 98.2% *n* = 4.3/21) and adherence (86% *n* = 18/21cf 86% *n* = 18/21)
	Oleske et al*.* 2007 [52]	Eligible workers were sent letters from the study principle investigator, plant management and local union official inviting them to the study. *N* = 454 participants joined, of which 79.3% were male (*n* = 360)	Overall 31% attrition in 12 months (*n* = 141 drop outs), 83.6% of male participants had complete data (*n* = 168/201), 75.9% of male participants were considered drop out or had missing data collection (*n* = 192/253)
	Ullman et al*.*1998 [56]	Mail surveys sent to participants with study information, a University pen and description of incentive once survey was sent back complete. Overall recruitment rate of 76% (*n* = 616/814; n.d for individuals identifying as male). 64.5% responded with no reminder (*n* = 334/518), 18.9% with one reminder (*n* = 98/518), 14.2% with two reminders (*n* = 74/518) and 2.3% with 3 reminders (*n* = 12/518) 32.6% of non-responders (*n* = 32/98) responded after receiving a second letter and $25 cheque A substudy of males demonstrated that 93% were retained (201/216). Of these 46.3% of males responded with reminders only (100/216) and a further 7.9% responded with reminders and a cheque (17/216)	Overall attrition rate: 10.7% (*n* = 550/616)
	Markanday et al*.* 2013 [58]	Letter of invitation contained information about the studies purpose, requirements of the participants and time investment required and where study centre was located. 67.1% recruitment rate for those who identified as male (*n* = 1,540/2,296), 77% for those identifying as female (*n* = 1,494/1,938)	n.d
	Méjean et al*.* 2014 [43]	6,556 participated from a total of 15,000 randomly invited participants from the NutriNet-Santé Study. 6,352/6,556 participants available for analysis. Of this sample 1,531 male (24.1%)	From those who dropped out of the substudy (*n* = 9,982; defined as participating stopping within 6 months after the inclusion in the cohort), 2,398 identified as male (24%)
**Purposeful Sampling**	Amin et al*.* 2023 [38]	From 187 PREVENT-ADPKD trial participants completed the questionnaire 78% (*n* = 146/187). Of this sample	Saturation for post-trial interview was reached at 74% (*n* = 108/146). A further 18.4% declined an interview (*n* = 7/38). Groups not stratified by sex
**Recruitment From Hospital or Research Centre**	Crichton et al*.* 2012 [57]	16.7% of total participants (*n* = 14/84) indicated an interest for future studies at Research Centre. Written advertisement also placed at local hospital n.d on percentages recruited	*Reported above*
	Gourash et al*.* 2022 [48]	n.d	2% (*n* = 50/2,458) attrition after year 1 assessment, 2.7% (*n* = 67/2,458) attrition after year 2, 3.5% (*n* = 80/2,458) attrition after year 3, 4.1% (*n* = 100/2,458) attrition after year 4, 4.9% (*n* = 121/2,458) attrition after year 5. Of which 0.4%, 0.7%, 1%, 1.2% and 1.7% was accounted by due to death, respectively. Groups not stratified by sex
	Janus et al*.* 1997 [51]	n.d	7.2% attrition before the first-year assessment (*n* = 15/209) and 28.2% (*n* = 59/209) after the first-year assessment (assumed between years 1–4). 35.4% lost over the 4-year study (*n* = 74/209)
	Kannisto et al*.* 2017 [44]	33.3% recruitment rate from eligible participants (*n* = 1,139/3,417). Of these, 49.2% (*n* = 560) who identify as male were randomised	4.8% attrition rate during the intervention period of all participants (*n* = 27/560) with 6 of the 27 withdrawals identifying as male (6/560; 1.1%). During a follow up postal survey 59% attrition rate who identify as male (*n* = 326/589)
	Pogue et al*.* 2022 [54]	*Recruitment rate reported above*. 920 participants had demographic characteristics. Of these, 76.4% (*n* = 703/920) were recruited via Digital Health Journey, of which 26.2% (*n* = 184/703) identified as male. Another 1.6% were recruited through study referral at site (*n* = 15/920), of which 53.2% identified as male (*n* = 8/15)	*Reported above*
	Schilling et al*.* 2023 [37]	99.2% of eligible individuals consented (*n* = 6,375/6,426). 92.63% (*n* = 5,905/6,375) of these individuals completed baseline surveys and of these participants, 74.02% identified as male (*n* = 4,371/5,905)	1.4% attrition rate from completion of baseline survey to COVID-19 analysis (*n* = 5,823/5,905). Groups not stratified by sex
**Recruitment Through Community Venues – Town centre, Workplaces, Community Groups, Football Grounds, Golf Clubs, Library, Shopping Centre**	Crichton et al*.* 2012 [57]	Written advertisements placed on noticed boards at libraries and shopping centres. n.d on percentages recruited	*Reported above*
	Irvine et al*.* 2017 [41]	Field visits through community venues recruited the last 52.9% of participants (*n* = 470 males approached). One participant was recruited for every 11 community venue visits	*Reported above*

### Barriers

A variety of factors were identified that interfered with male participation in longitudinal research are shown in Table 4. Some of these were situational and included participant death or relocation [36, 39, 42, 45, 48, 51, 53, 55, 57, 61]. While other barriers included time commitment [40, 58], reluctance for medical testing [58], or the belief that the study is an invasion of privacy [58]. A large number of studies reported that men did not attend study visits [40, 58], were not interested in the study or could not be bothered to participate [36, 40, 41, 44, 48, 49, 51, 58, 61], and study staff received no response to invitations [40, 41, 61].

**Table 4:** Barriers to participation and drop-out or refusal rates of participants

*Reasons for refusal to participate or drop-out*	*Proportion of participants who refuse participation*	*References*
*Appointment non-attendance*	14.0% of passive refusals	[40]
	2.1% of males that refused to participate	[58]
	1.3% of males were unable to cope with study requirements due to old age	[58]
*Comprehensio*n* of the study requirements*	0.4% of males refused to participate	[58]
*Cannot be bothered/not interested*	27.8% of active refusals	[40]
	13.8% declined to participate	[41]
	66.7% refused to participate	[44]
	39.6% males that refused to participate	[58]
	18.5% of eligible participants	[49]
*Did not meet inclusion criteria*	87% of eligible participants	[49]
*Time commitment*	38.9% of active refusals	[40]
	26.3% of males that refused to participate	[58]
*Invasion of privacy*	0.3% of males that refused to participate	[58]
*Medical*	15.5% of participants who refused to participate	[57]
	35.6% of participants unable to attend due to illness, 0.2% of participants had limited medical information	[40]
	16.9% of males that refused to participate	[58]
*Unable to contact/no response*	35.0% of eligible participants	[40]
	17.4% of eligible participants	[41]
	40.7% of invited male participants	[61]
*Psychopathology factors*	0.8% of males refused to participant in case a medical problem was uncovered	[58]
*Reluctance over medical testing*	1.1% of males that refused to participate	[58]
*Religious/philosophical reasons*	0.1% of males that refused to participate	[58]
*Third party involvement*	62.2% of participants passively refused via a relative, 15.9% of participants passively refused by resident/nursing home	[40]
	18.4% transferred to another ward or discharged from hospital or research nurse forgot to ask	[44]
*Unknown reason/personal reason*	5.2% of males that refused to participate	[58]
	9.5% of eligible participants in 1968	[45]
	28.6% of active refusals	[40]
	3.2% of eligible participants	[41]
	3.6% of eligible participants	[49]
	***Proportion of participants who were non-completers/Further suggestions for improvements by completing participants***	
*Appointment non-attendance*	3.2% of non-completers	[41]
	24.7% missed at least one visit by end of study (12 months)	[52]
	Quantitative data- in person visits were difficult to attend due to the distance of the centre	[53]
*Communication*	Qualitative data- better coordination of communication for study results to participants	[38, 49]
	Qualitative data- increased personalisation would increase engagement like a personal question the participants could contemplate over the next week	[49]
	Qualitative data- increase of data sharing between research team, treatment therapist and each participant would have increased engagement and data tracking over the period	[49]
*Education*	Qualitative data- Increasing the education around the condition that is the focus of the trial	[38]
*Medical*	12.7% non-completers	[57]
	4.1% of non-completers had a child that had an additional diagnosis	[51]
	Qualitative data- state of the participants personal health and the nature of the intervention may affect future enrolment	[38]
*Situational (lack of reliable housing, moving, death)*	1.4% of non-completers	[57]
	95.2% of non-completers	[42]
	1.4% of non-completers from wave 1 (1974) to 47.3% in wave 5 (2011)	[45]
	6.8% to 30.6% of non-completers across 6 different centres	[61]
	25.7% of non-completers moved, 5.4% of families had a child who died	[51]
	21.4% of non-completers died, 17.4% moved away	[36]
	2.3%—9.4% of non-completers (wave 1–5)	[39]
	Qualitative data- unable to complete exercise or have appropriate meal preparation	[53]
	63.4% of non-completers	[55]
	35.5% of non-completers died	[48]
*Inability to adhere to study activities*	Qualitative data- unable to complete training due to unreliable technology	[53]
	12.7% of non-completers	[57]
	10.1% did not receive allocation of intervention	[41]
	1.7% of non-completers did not like research assessment, 0.8% of non-completers were incarcerated	[48]
*Cannot be bothered/ loss of interest/wanted to withdraw*	40.8% of non-completers	[36]
	9.5% of non-completers	[51]
	3.2% of non-completers	[44]
	20.6% of non-completers	[61]
	13.2% of non-completers	[48]
*Difficulty to arrange follow-up appointments with participants*	6.8% of non-completers	[51]
	20.6% of non-completers	[48]
*Missing data/incomplete data*	6.5% of participants	[41]
	52.5% of participants did not complete the final postal survey, 0.36% of participants did not have available data in the Finnish national Care Register for Health Care	[44]
	15.0% of non-completers	[61]
	3.1% of participants	[43]
*Time commitment*	5.6% of non-completers	[57]
	17.6% of non-completers	[51]
	Qualitative data- competing demands in personal life, unable to prioritize program participation	[53]
	Qualitative data- 24-h urine output collection during work hours was difficult and restrictive, taking days of work and losing wages	[38]
*Lost contact*	8.5% of non-completers	[57]
	27.8% of non-completers	[44]
	1.7% of non-completers	[48]
*Unknown reason/personal reason*	7.0% of non-completers	[57]
	4.8% of non-completers	[42]
	4.8% of non-completers	[44]
	21.6% of non-completers	[51]
	20.9% of non-completers from wave 1 (1974) to 24.1% in wave 5 (2011)	[45]
	10.7% of non-completers	[56]
	57.5% of non-completers were lost by 1-year follow up	[46]
	16.5% of non-completers	[48]
*Difficulty in comprehending the study*	2.7% of non-completers	[51]
	Qualitative data- reducing length and complexity of questionnaires and understanding the potential risks	[38]
*Psychopathology factors*	Paranoid factor had an elevated but non-significant risk for early drop out (26.9%) Dysphoric Borderline factor put a significant risk for late dropout (15.9%)	[50]
	Qualitative data- side effects from medications for mental health or chronic pain were issues in completing the program. Social anxiety of talking openly to other participants also prohibited some participants interaction	[53]
*Third party involvement*	6.8% of non-completers – due to family issues	[51]
	Qualitative data- consider withdrawing when family was sick	[38]
	4.2% of non-completers—Work and family responsibilities	[48]
*Financial Hardship*	Qualitative data- participants across all treatment groups found recommendations of what to eat and how to exercise cost prohibitive	[53]
	Qualitative data- providing monetary incentives	[38]
*Technical Issues*	Qualitative data- difficulties in troubleshooting web-based program after logging in as well as printing physical activity log	[53]

### Facilitators

Many studies employed a variety of strategies to increase participation for males (Table 5). These varied from offering free medical screening [36], reminders for appointments [40, 42, 46, 48, 51, 52, 56–58, 61], or enrolment of wives [36] or family members [39, 51] to assist in retention. Several studies used a range of strategies, particularly [43, 56, 57], with varying degrees of success.

**Table 5:** Facilitating factor that improved recruitment and retention of males

*Facilitating factor or approach*	*Study details (for either sex)*	*Study details for male specific approaches*
*Advertising through mainstream media, recurrent attention from medical press and general media*	[42, 43, 55, 57]	[61]
*Annual Cohort Symposium*	[42]	-
*Clinical coordinator referrals*	[37]	-
*Continuity of care with the same research staff*	-	[38]
*Delivery of intervention using friendly, relaxed, non-directive style with easily understood information*	-	[41]
*Delivery of GIFS after assessment were beneficial providing humour and personalisation*	[49]	-
*Development of loyal, close relationships in person or one on one check-ins over the phone*	In person: [38, 51] Telephone: [38, 53]	
*Discussion of reasons for refusal, drop out, or non-attendance*	[40, 44, 51, 58]	[61]
*Enrolment of spouse or all family members*	[39, 51]	[36]
*Free medical screening/laboratory test results and clinical report*	[43, 46, 48, 57]	[36]
*Incentives/Reimbursement*	Monetary: [46–49, 56, 57] Paid parking: [48, 51] Small gifts: [43, 48, 51, 52, 56] Snacks during assessment: [48]	-
*Invitation to press conferences*	[43]	-
*Maintaining regular contact using one or several methods of contact*	[44, 48, 51, 57]	[41]
*Membership card to study and certificate of completion at each follow-up*	[43]	-
*Participant choice in amount, timing and frequency of participant intervention or location of interview/ Convenien*ce* of time/location*	Timing and frequency: [44], Time and location of interview: [40] Convenient timing and/or location: [58] Convenient location including home assessment, options for less burdensome data collection or combined with clinical visit: [48]	Convenient timing and/or location: [36, 41]
	Multiple geographical locations, low frequency of the procedures and flexible appointments: [38]	
*Participant newsletters*	Yearly newsletter written from PI: [42] Annual holiday letter: [51] Monthly email with scientific information about health/nutrition: [43] Study result newsletters, birthday, and holiday cards: [48]	Birthday and Christmas cards: [61] Study Newsletter: [36]
*Participant perceptions*	Aiding research particularly publicly funded research: [43], helping family and friends with COVID-19 and advancing research: [37], knowledge gain and treatment benefit: [38]	Perceived health benefits: [36, 41] Satisfaction at being part of ‘health elite’ (Hawthorne Effect): [36]
*Participant website*	[48]	
*Payment of wages to employers to attend study visit without loss of income or work penalty*	[52]	[36]
*Personal notes to participants*	[56]	-
*Prompting participants to update changes in address or phone number by SMS, post or prior to surgery*	Pre-surgery [48]	SMS: [41] Post: [61]
*Referrals by family and friends to the study*	[37]	-
*Reminders for questionnaires/ appointments or follow up after non-attendance/non-responders*	[40, 42, 46, 48, 51, 52, 56–58]	[61]
*Recruitment *via* “mass mailing”*	[55]	-
*Resources specific to intervention*	[40, 57, 58]	[41, 61]
*Sensitive and informative communication by research staff/ participants or partners of participants to meet individually*	Sensitive communication style: [38]	Individual (participant or partner of participant instead of group settings: [50]
*Screening participants from geographical stable workplaces*	-	[36]
*Simple consent process*	Online: [46] Verbal and Written: [38]	Via SMS: [41]
*Study data collected completely online*	[43, 57]	-
*Vested personal interest*	Desire to contribute to chronic disease risk: [43], Desire to help country and learning how to protect health: [37], Desire to help others: [38], No other medical options available: [38]	Desire to contribute their own weight loss: [41], Desire to help others with disease and for self: [41], Desire for education about chronic disease: [38, 41]
*Written Questionnaires*	[38]	

## Discussion

We have undertaken a thorough assessment of longitudinal studies to determine recruitment and retention facilitating strategies for male participants. Retention of male participants was particularly impressive in two studies at 85.5% (from *n* = 69 recruited) [41] and 88.9% (from *n* = 2,280 recruited) [36] over a period of 5 months and 12 years, respectively. Irvine et al., undertook a trial to reduce alcohol related consumption to reduce obesity and relied upon the perceived health benefits for their participants [41], while Rose et al., studied ageing in Veteran participants. Rose et al., retained participants over 12 years, through a diverse range of approaches including; the use of participant newsletters, short study visits, free medical screening, income supplementation, encouraged participant perceptions of being part of the ‘health elite’ and recruited wives to assist with retention [36]. This study began in 1976 where there was a greater inclination by the public to follow suggestions, also particularly true for their target Veteran population [36]. Like Rose et al. [36], the Irvine et al., study team maintained regular contact, ensuring convenient timing and location of study visits and continued to highlight perceived health benefits of the research project. The Irvine et al., research team spent additional time ensuring that their staff conversations and project literature was relaxed and friendly and non-directive in its approach [41]. While Rose et al., provided re-imbursement to employers for study attendance, neither of these top two studies [41] used a direct financial or gift incentive to participants but rather relied upon excellent communication strategies.

Several studies highlight that different aged males are retained at different rates in their studies. For example, Cheraghi et al., saw 67% 40–59 year males retained while only 55% of > 60 years in the same study were retained [39]. In the male only studies, Hamberger et al. [50], and Lee et al. [61], showed varied retention rates based upon age of the participants. Male only studies have shown that diverse approaches can be successful in recruitment and retention. Communication that is non-directive in style, clear and delivered by supportive staff was important for Irvine et al. [41]. Continuing to maintain contact with male participants was important and included contact through family or a spouse [36], Christmas [61] and birthday cards, and study related newsletters [36]. Male only studies have highlighted that where male participants have a vested interest, for example, weight loss and desire for health education, these interests can be important drivers for recruitment [41].

### Barriers to recruitment and retention of males

Barriers varied and were related to an inability of participants to participate due to lack of understanding of study objectives [58], language barriers [58] or lack of access to the internet for studies being conducted online [53]. Table 4 highlights reasons given for refusal to participate and reasons for non-completion of a study.

In intervention studies focused on lifestyle changes, barriers to participation included inability to adhere to the study activities [57] or lack of motivation to engage with new technologies [44]. In one study where participants had to make dietary changes and frequently visit the research centre, participants expressed frustration in trying to implement study content due to associated financial costs involving more expensive food, transportation or computer access [53]. Participant feedback included that transportation and free meal options would have been more enticing [44]. Travelling to the study centre was found to be a barrier in two studies [53] and another study participants expressed a preference for study to be online [53]. Another barrier reported in two studies was difficulty in arranging a follow-up session [53].

The major causes for refusal or dropout were time commitment issues and lack of interest in the study [40, 44, 51, 58]. Time commitment issues were related refusal to having to make frequent visits to the study centre [51, 57, 58] and lifestyle changes that required more time, such as exercising or taking time to cook meals [53]. Health issues played a role in participant attrition and participants with health issues [42, 51, 53, 57, 58] and psychological issues [50] were most likely to refuse participation or dropout.

Different demographic characteristics were associated with refusal to join a study or a particular data collection point. These included low socioeconomic status [40, 42], younger age [40, 42–44, 52], older age [40, 61], poor lifestyle factors [42] and being unmarried [43, 45]. In a birth cohort study, non-participation was linked to fathers being born outside the country where the study resided or having lower education [51].

Interestingly, Ullman et al., explored factors related to types of study responders; (non-responders, reluctant responders and responders) in an ongoing longitudinal study. Findings demonstrated that males who considered themselves more attractive or having better relationships with others were more likely to respond, while those that felt worse about their own sense of self required more incentives and reminders in order to take part in the study [56].

### Facilitators to recruitment and retention of male participants

While many of the facilitators listed in Table 5 would be suitable for either sex, using a male-centric approach would likely prove particularly useful. Study advertising on mainstream media or medical press was used as a method to establish study credibility [42, 57]. Several studies maintained contact with their participants throughout the study [42, 57]. In one study, a yearly letter, written by the principal investigator, was sent to participants [42], other studies sent out a study newsletter [36] or monthly emails with health and nutritional scientific information [43]. Two studies sent an annual holiday letter [51] and in another participants received birthday and Christmas cards [61]. These methods were thought to pique participant interest and motivate them to participate in study activities. Interestingly, Griffith Filipo et al., used humour with participants through the use of humourous GIFs sent to participants following study visits and found these to be a facilitating factor [49].

Other motivational techniques for participation included increasing study accessibility and minimising interference with participants day-to-day activities. For example, one of the studies was designed to ensure that examinations only took a couple of hours [36]. Another study was designed to be exclusively online which was a determining factor for participation in 46.45% of the sample [43]. In a trial where participants had to answer SMS messages, participants were able to choose the amount, timing, and frequency of texts they received, with the ability to change these options throughout the study course [44]. Two studies planned with employers to pay participants regular wages or give leave without penalty while they participated in the study [43]. Finally, one study reported that additional interventions were implemented for people that struggled to adhere to the required activities [55].

Incentives were successful in study participation. Six studies gave monetary incentives [46–49, 56, 57]. Other studies gave participants small gifts such as membership cards, certificates of completion, pens, tee shirts, mugs, etc. [56, 57]. In an intervention study where participants had to consume specific products, these products were provided freely for participants [57]. A few health interventions offered free medical screenings [43, 46, 57]. Participants in the Rose et al., were notified of the outcome of their medical examinations and alerted if anything was abnormal, which in some cases prevented life-threatening issues [36]. To minimise attrition, participant reminders to complete questionnaires or arrange appointments in multiple studies [40, 42, 46, 52, 56, 57, 61]. One study found that when participants were contacted to assess reasons for refusal this prompted some to change their minds and participate in the study [40, 42, 46, 52, 56, 57, 61].

One aspect that was associated with male participant retention were the perceived health benefits gained from participating in the study [36, 41, 57]. One study specifically highlighted its participants expressed satisfaction of being part of a *“health elite”*, which was associated with high retention rates [36]. Another beneficial factor was the idea that their involvement in the study aided research in the field of nutrition (22.24%) and advanced public health (61.37%) [43]. More recently this has been shown to be true during the COVID-19 pandemic where males participated in high rates [36, 41, 57]. Méjean et al., reported that 67.02% of participants expressed satisfaction that the study was funded exclusively by public sources which was perceived as unbiased, and this was particularly well received by male participants [43]. Finally, in an attempt to motivate male participants, Rose et al., enlisted participants’ wives in the study, which was found to have positive outcomes in retention [36].

The greatest challenge for data extraction for this review was the way in which authors report these figures in their studies. Many studies report on overall recruitment, retention and barriers but few studies clearly incorporated sex-specific findings. Interests, and drivers for behaviour are unique between sexes and therefore it is important that research projects report separated male and female specific findings. The most beneficial studies reviewed gave recruitment success with each approach, for example Crichton et al. [57], highlighted what number of participants were recruited from varied strategies including advertising via TV or newspaper, letter of invitation, from the hospital, or a notice in the library. The Ullman et al. [56], study was also clear in highlighting how many approaches they needed to have data returned to them, for example, immediately, after one reminder, or multiple reminders and a financial incentive. Likewise, studies who reported when or how they noticed attrition for their research were incredibly valuable [42, 44, 46, 51, 57, 61].

### Strengths and limitations

The strengths of this systematic review lie in its comprehensive compilation of research data from the past 47 years of male recruitment and retention in longitudinal research studies which has historically posed many issues to researchers [5, 10–19]. To the authors’ knowledge at the time of print, there is no other systematic review available on the barriers and facilitators of the recruitment and retention of males in longitudinal research. It has been evident that the barriers and facilitators are not unique to a specific study aim but have been experienced across the diverse range of studies. This systematic review offers a comprehensive list of strategies which have worked with particular populations and strategies which have failed for researchers looking to improve their male recruitment and retention rates and has a particular focus on longitudinal research studies. Primarily it has highlighted that multiple facilitators will be needed when designing longitudinal research inclusive of males, as the barriers to participation are diverse. The most challenging barrier to overcome is how to develop enthusiasm and urgency from men towards health research. Regardless of the purpose of the underlying study, the barriers and facilitators for male participants are relatively consistent.

The exclusion of several population groups limits this study however it was felt that each of these required a detailed separate systematic review to ensure that the unique barriers and facilitators for the recruitment and retention of these communities are clearly articulated. A further limitation is that for papers to be included in this systematic review, they had to specifically mention an issue that detailed barriers/facilitators to recruitment/retention in the title/abstract rather than stating *“we recruited”* in the full text. Therefore, we acknowledge there it may be possible that some publications that focused on longitudinal studies involving male participants have been missed. In conclusion, this systematic review offers an in-depth look into the barriers and facilitators of the recruitment and retention strategies for males aged 17–59 years old for the past 47 years. It highlights that research teams will need to expend considerable time, expense and diverse approaches to successfully engage and retain male participants into longitudinal studies.

### Supplementary Information

**Additional file 1.** Final Search Stategies.

## Data Availability

All data generated or analysed during this systematic review are included in this published article.
