# Supplementary material for: Barriers and facilitators for recruiting and retaining male participants into longitudinal health research: a systematic review
Source: BMC Med Res Methodol. 2024 Feb 22;24:46. doi: 10.1186/s12874-024-02163-z (PMC10882922; doi:10.1186/s12874-024-02163-z)
Supplement: Supplementary file 1 — Additional file 1. Final Search Stategies. [file 12874_2024_2163_MOESM1_ESM.docx]

**Supplementary File 1**

**Final Search Stategies**

# Ovid MEDLINE

(

((engag* OR keep* OR maintain* OR motivat* OR enable* OR support* OR boost* OR facilitat* OR increase OR increased OR increases OR increasing OR encourag* OR maximis* OR maximiz* OR promot* OR improv* OR incentiv* OR barrier* OR inhibit* OR withhold* OR obstacle* OR obstruct* OR challeng* OR problem* OR disincentive* OR miminis* OR miminiz* OR prevent* OR lessen OR decreas* OR reduc* OR decline OR declines OR declining OR influenc* OR affect* OR effect* OR impact* OR determin* OR predict* OR alter OR alters OR altering)

ADJ3

(recruit* OR retain* OR retention OR participat* OR involve OR involved OR involving OR involvement OR enrolling OR enrollment* OR enrolment* OR non-participa* OR non-response* OR response rate* OR loss to follow-up OR loss to followup OR attrition OR drop out* OR dropout* OR drop-out* OR withdrawal* OR compliance OR noncompliance OR non-compliance)

ADJ3

(study OR studies OR research OR health research* OR trial* OR survey* OR longitudinal OR cohort OR panel stud* OR prospective OR long term stud* OR long term research OR long term trial* OR longer term stud* OR longer term research OR longer term trial* OR extended stud* OR extended research OR extended trial* OR clinical trial* OR controlled trial* OR follow-up stud* OR follow-up research OR follow-up trial* OR followup stud* OR followup research OR followup trial*)).ti,ab.

OR

((strategy OR strategies OR method OR methods OR plan OR plans OR intervention*) ADJ3 (recruitment OR recruiting OR recruit OR retention)).ti,ab.

OR

((recruitment OR (retention NOT (urinary retention OR retention in care OR weight retention OR prosthesis retention OR knowledge retention)) OR loss to follow-up OR loss to followup OR attrition OR drop out* OR dropout* OR drop-out*)

AND

(study OR studies OR research OR trial* OR survey* OR cohort)).kf.

)

AND

((longitudinal OR long term OR longer term OR panel stud* OR repeat measures OR repeated measures OR follow-up study OR follow-up studies OR followup study OR followup studies).ti,ab.
OR ((cohort OR prospective OR extended OR nested) ADJ3 ((study OR studies OR research OR health research* OR trial* OR survey*)).ti,ab.
OR Longitudinal Studies/ OR Cohort Studies/ OR Follow-Up Studies/ OR Prospective Studies/))
NOT (exp animals/ not humans.sh.)

NOT (review.pt. OR systematic review.pt. OR case reports.pt.)

limit 1 to english language

*Comments on search strategy*

- The following MeSH terms were considered, but not included:
  - Patient Selection/ - unfortunately does not differentiate between selection for treatment and selection for research participation
  - Refusal to Participate/ - broader than participation in research, includes refusal to participate in medical procedures or health promotion programs
  - Patient Dropouts/ - focuses on dropout from health care, not research participation
  - Research Subjects/ - while this term does represent the concept of research subjects, it is broader than any of the concepts useful in this particular search
  - Patient Participation/ - focuses on participation in health care, not research participation
  - Lost to Follow-Up/ - even though the term looks like it's to do with cohort studies, a lot of the articles tagged with it were about loss to treatment followup
- This was a difficult search as many of the terms representing the key concepts are also generic terms that could be found in many irrelevant papers, eg. study, recruit, strategy, intervention, cohort etc. The language describing the concepts of recruitment/retention barriers, facilitators and strategies is also not very standardised. The search strategy balances the desire for comprehensiveness with the need to not retrieve a very large proportion of irrelevant results. The search was repeatedly tested while being developed to judge the effects of decisions made.

# Embase (Embase.com)

(

((engag* OR keep* OR maintain* OR motivat* OR enable* OR support* OR boost* OR facilitat* OR increase OR increased OR increases OR increasing OR encourag* OR maximis* OR maximiz* OR promot* OR improv* OR incentiv* OR barrier* OR inhibit* OR withhold* OR obstacle* OR obstruct* OR challeng* OR problem* OR disincentive* OR miminis* OR miminiz* OR prevent* OR lessen OR decreas* OR reduc* OR decline OR declines OR declining OR influenc* OR affect* OR effect* OR impact* OR determin* OR predict* OR alter OR alters OR altering)

NEAR/3

(recruit* OR retain* OR retention OR participat* OR involve OR involved OR involving OR involvement OR enrolling OR enrollment* OR enrolment* OR non-participa* OR non-response* OR "response rate*" OR "loss to follow-up" OR "loss to followup" OR attrition OR "drop out*" OR dropout* OR drop-out* OR withdrawal* OR compliance OR noncompliance OR non-compliance)

NEAR/3

(study OR studies OR research OR "health research*" OR trial* OR survey* OR longitudinal OR cohort OR "panel stud*" OR prospective OR "long term stud*" OR "long term research" OR "long term trial*" OR "longer term stud*" OR "longer term research" OR "longer term trial*" OR "extended stud*" OR "extended research" OR "extended trial*" OR "clinical trial*" OR "controlled trial*" OR "follow-up stud*" OR "follow-up research" OR "follow-up trial*" OR "followup stud*" OR "followup research" OR "followup trial*")):ti,ab

OR

((strategy OR strategies OR method OR methods OR plan OR plans OR intervention*) NEAR/3 (recruitment OR recruiting OR recruit OR retention)):ti,ab
OR

(
(recruitment OR (retention NOT ("urinary retention" OR "retention in care" OR "weight retention" OR "prosthesis retention" OR "knowledge retention")) OR "loss to follow-up" OR "loss to followup" OR attrition OR "drop out*" OR dropout* OR drop-out*)

AND

(study OR studies OR research OR trial* OR survey* OR cohort)
):kw

)

AND

((longitudinal OR "long term" OR "longer term" OR "panel stud*" OR "repeat measures" OR "repeated measures" OR "follow-up study" OR "follow-up studies" OR "followup study" OR "followup studies"):ti,ab

OR ((cohort OR prospective OR extended OR nested) NEAR/3 (study OR studies OR research OR "health research*" OR trial* OR survey*)):ti,ab
OR 'Longitudinal Study'/exp OR 'Cohort Analysis'/de OR 'Prospective Study'/de)

NOT ('animal'/exp NOT 'human'/exp)

NOT (review:it)
AND [embase]/lim
AND [english]/lim

# CINAHL (EBSCO)

(

((TI engag* OR AB engag* OR TI keep* OR AB keep* OR TI maintain* OR AB maintain* OR TI motivat* OR AB motivat* OR TI enable* OR AB enable* OR TI support* OR AB support* OR TI boost* OR AB boost* OR TI facilitat* OR AB facilitat* OR TI increase OR AB increase OR TI increased OR AB increased OR TI increases OR AB increases OR TI increasing OR AB increasing OR TI encourag* OR AB encourag* OR TI maximis* OR AB maximis* OR TI maximiz* OR AB maximiz* OR TI promot* OR AB promot* OR TI improv* OR AB improv* OR TI incentiv* OR AB incentiv* OR TI barrier* OR AB barrier* OR TI inhibit* OR AB inhibit* OR TI withhold* OR AB withhold* OR TI obstacle* OR AB obstacle* OR TI obstruct* OR AB obstruct* OR TI challeng* OR AB challeng* OR TI problem* OR AB problem* OR TI disincentive* OR AB disincentive* OR TI miminis* OR AB miminis* OR TI miminiz* OR AB miminiz* OR TI prevent* OR AB prevent* OR TI lessen OR AB lessen OR TI decreas* OR AB decreas* OR TI reduc* OR AB reduc* OR TI decline OR AB decline OR TI declines OR AB declines OR TI declining OR AB declining OR TI influenc* OR AB influenc* OR TI affect* OR AB affect* OR TI effect* OR AB effect* OR TI impact* OR AB impact* OR TI determin* OR AB determin* OR TI predict* OR AB predict* OR TI alter OR AB alter OR TI alters OR AB alters OR TI altering OR AB altering)

N2

(TI recruit* OR AB recruit* OR TI retain* OR AB retain* OR TI retention OR AB retention OR TI participat* OR AB participat* OR TI involve OR AB involve OR TI involved OR AB involved OR TI involving OR AB involving OR TI involvement OR AB involvement OR TI enrolling OR AB enrolling OR TI enrollment* OR AB enrollment* OR TI enrolment* OR AB enrolment* OR TI non-participa* OR AB non-participa* OR TI non-response* OR AB non-response* OR TI "response rate*" OR AB "response rate*" OR TI "loss to follow-up" OR AB "loss to follow-up" OR TI "loss to followup" OR AB "loss to followup" OR TI attrition OR AB attrition OR TI "drop out*" OR AB "drop out*" OR TI dropout* OR AB dropout* OR TI drop-out* OR AB drop-out* OR TI withdrawal* OR AB withdrawal* OR TI compliance OR AB compliance OR TI noncompliance OR AB noncompliance OR TI non-compliance OR AB non-compliance)

N2

(TI study OR AB study OR TI studies OR AB studies OR TI research OR AB research OR TI "health research" OR AB "health research" OR TI trial* OR AB trial* OR TI survey* OR AB survey* OR TI longitudinal OR AB longitudinal OR TI cohort OR AB cohort OR TI "panel stud*" OR AB "panel stud*" OR TI prospective OR AB prospective OR TI "long term stud*" OR AB "long term stud*" OR TI "long term research" OR AB "long term research" OR TI "long term trial*" OR AB "long term trial*" OR TI "longer term stud*" OR AB "longer term stud*" OR TI "longer term research" OR AB "longer term research" OR TI "longer term trial*" OR AB "longer term trial*" OR TI "extended stud*" OR AB "extended stud*" OR TI "extended research" OR AB "extended research" OR TI "extended trial*" OR AB "extended trial*" OR TI "clinical trial*" OR AB "clinical trial*" OR TI "controlled trial*" OR AB "controlled trial*" OR TI "follow-up stud*" OR AB "follow-up stud*" OR TI "follow-up research" OR AB "follow-up research" OR TI "follow-up trial*" OR AB "follow-up trial*" OR TI "followup stud*" OR AB "followup stud*" OR TI "followup research" OR AB "followup research" OR TI "followup trial*" OR AB "followup trial*"))

OR

((TI strategy OR AB strategy OR TI strategies OR AB strategies OR TI method OR AB method OR TI methods OR AB methods OR TI plan OR AB plan OR TI plans OR AB plans OR TI intervention* OR AB intervention*) N2 (TI recruitment OR AB recruitment OR TI recruiting OR AB recruiting OR TI recruit OR AB recruit OR TI retention OR AB retention))

)

AND

(

(TI longitudinal OR AB longitudinal OR TI "long term" OR AB "long term" OR TI "longer term" OR AB "longer term" OR TI "panel stud*" OR AB "panel stud*" OR TI "repeat measures" OR AB "repeat measures" OR TI "repeated measures" OR AB "repeated measures" OR TI "follow-up study" OR AB "follow-up study" OR TI "follow-up studies" OR AB "follow-up studies" OR TI "followup study" OR AB "followup study" OR TI "followup studies" OR AB "followup studies"

OR

((TI cohort OR AB cohort OR TI prospective OR AB prospective OR TI extended OR AB extended OR TI nested OR AB nested) N2 (TI study OR AB study OR TI studies OR AB studies OR TI research OR AB research OR TI "health research*" OR AB "health research*" OR TI trial* OR AB trial* OR TI survey* OR AB survey*)))

OR MH "Prospective Studies+" OR MH "Repeated Measures")

NOT ((MH animals+ OR MH animal studies OR TI (animal model*)) NOT MH human)
NOT (PT review OR PT systematic review)
AND LA english

# Web of Science (Clarivate Analytics)

(

((engag* OR keep* OR maintain* OR motivat* OR enable* OR support* OR boost* OR facilitat* OR increase OR increased OR increases OR increasing OR encourag* OR maximis* OR maximiz* OR promot* OR improv* OR incentiv* OR barrier* OR inhibit* OR withhold* OR obstacle* OR obstruct* OR challeng* OR problem* OR disincentive* OR miminis* OR miminiz* OR prevent* OR lessen OR decreas* OR reduc* OR "decline" OR "declines" OR "declining" OR influenc* OR affect* OR effect* OR impact* OR determin* OR predict* OR "alter" OR "alters" OR "altering")

NEAR/2

(recruit* OR retain* OR "retention" OR participat* OR involve OR involved OR involving OR involvement OR "enrolling" OR enrollment* OR enrolment* OR non-participa* OR non-response* OR "response rate*" OR "loss to follow-up" OR "loss to followup" OR "attrition" OR "drop out*" OR dropout* OR drop-out* OR withdrawal* OR "compliance" OR "noncompliance" OR "non-compliance")

NEAR/2

("study" OR "studies" OR "research" OR "health research*" OR trial* OR survey* OR "longitudinal" OR "cohort" OR "panel stud*" OR "prospective" OR "long term stud*" OR "long term research" OR "long term trial*" OR "longer term stud*" OR "longer term research" OR "longer term trial*" OR "extended stud*" OR "extended research" OR "extended trial*" OR "clinical trial*" OR "controlled trial*" OR "follow-up stud*" OR "follow-up research" OR "follow-up trial*" OR "followup stud*" OR "followup research" OR "followup trial*"))

OR

(("strategy" OR "strategies" OR "method" OR "methods" OR "plan" OR "plans" OR intervention*) NEAR/2 ("recruitment" OR "recruiting" OR "recruit" OR "retention"))

)

AND

("longitudinal" OR "panel stud*" OR "repeat measures" OR "repeated measures" OR "follow-up study" OR "follow-up studies" OR "followup study" OR "followup studies" OR (("cohort" OR "prospective" OR "long term" OR "longer term" OR "extended" OR "nested") NEAR/2 ("study" OR "studies" OR "research" OR "health research*" OR trial* OR survey*)))

AND

LANGUAGE: (English) AND DOCUMENT TYPES: (Article OR Abstract of Published Item OR Correction OR Correction, Addition OR Discussion OR Early Access OR Editorial Material OR Letter OR Meeting Abstract OR Meeting Summary OR Note OR Proceedings Paper OR Reprint OR Retracted Publication OR Retraction)

Indexes=SCI-EXPANDED, SSCI, CPCI-S, CPCI-SSH, ESCI Timespan=All years
